# Experience of focused workshop intervention in presentation skills - Importance of foundational skills for ophthalmologists in training

**DOI:** 10.12688/mep.20114.2

**Published:** 2024-05-29

**Authors:** Snigdha Snigdha, Avinash Pathengay, Shefali Pandey, Ruby Kala Prakasam, Shobha Mocherla

**Affiliations:** 1 Academy for Eyecare Education, L V Prasad Eye Institute, Hyderabad, Telangana, 500034, India

**Keywords:** presentation skills, communication skills, soft skills, ophthalmology education, workshop intervention, self-rating questionnaire

## Abstract

**Background:**

The study was conducted to assess the impact of a workshop intervention designed to enhance presentation skills of ophthalmology fellows in training.

**Methods:**

A 10-hour workshop on presentation skills was conducted for ophthalmology fellows in the 2022 cohort at a single institution. An email was sent to the 2022 cohort to recruit participants. A total of 29 fellows (19 females, 10 males) volunteered to participate in the study. Participants completed a self-rating questionnaire to assess improvement in their presentation skills at four different time points of the workshop. The self-rating questionnaire utilized a ten-point rating scale (1–10) and evaluated properties and content (PC) and soft skills (SS). Data were analysed using SPSS software. Friedman and post-hoc tests compared self-ratings at four time points. Statistical significance was set at p-value < 0.05.

**Results:**

Both properties & content (PC) and soft skills (SS) showed significant difference (p < 0.001) post workshop compared to earlier stages. The higher self-rating (PC4 and SS4) highlights increased awareness towards the scope of improving the presentation after the workshop intervention.

**Conclusions:**

Presentation skills empower medical professionals to better communicate with diverse audiences, demonstrating their currency in medical knowledge, lobbying for correct understanding, and bringing praxis to pedagogy. The findings support the integration of similar workshops into medical curricula to foster well-rounded medical professionals.

## Introduction

Skilful communication is intrinsic to professionalism ^1^. For doctors, professional presentation skills are fundamental resources for interpersonal and public interaction. With professional aspirations increasingly linked to effectively managing impressions ^2^, clinicians, scientists and researchers are using every opportunity to present themselves well.

For professional presenters, presentation skills give shape to an idea, helping to share ongoing learning with multiple teams. For medical professionals speaking to diverse audiences – colleagues, peers, patients, and the public – presentation skills help them to demonstrate their currency in medical knowledge and lobby for staged skill transfer through correct understanding, recall, internalization, and implementation ^3^.

Effective communication makes the good doctor popular, trusted ^4, 5^. Soft skills for medical doctors are coveted assets, and include speaking in the language preferred by the patient, giving precise instructions to follow, showing genuine concern, and maintaining pleasant interactions ^6, 7^. Generally, doctors in training learn standard responses and behaviours by observing their peers and seniors ^8– 10^. In medical schools in India, students are not tutored in role play and patient interactions as curricular activities ^11, 12^. Inculcating soft skills in medical education will empower doctors to master their delivery of care and service ^13– 15^. Presentation skill development may be effected by staged skill transfer, and therefore was taken on as an initiative at an eye health network in south central and eastern central India.

## Methods

### Workshop genesis and details

A 10-hour long physical workshop on presentation skills development was conducted on 2 ^nd^ July 2022 for fellows under training pursuing an ophthalmology fellowship. All the participants (29) were recruited as fellows under a common session of ophthalmology fellowship and was the eligibility criteria for enrolment in the workshop. The workshop was conducted in a classroom setting where the facilitator could facilitate the activity with the help of required audio-visual assistance. The learnings acquired when conducting virtual workshops during the COVID-19 lockdown helped us improve the structure and quality of the physical workshop.

Workshop design: The workshop commenced with a series of short presentations delivered by each participant. This initial exchange established a foundation of shared experience and diverse perspectives. The moderator, actively engaged in the organization's academic pursuits, then presented a curated series of six presentations. Each one provided insights into various facets of crafting a compelling presentation. Following each presentation, a facilitated discussion allowed for in-depth exploration of the content.

The workshop's core resided in the lively, moderator-guided discussions amongst the participants. This emphasis on peer interaction fostered a dynamic exchange of ideas and insights. Furthermore, the workshop incorporated a self-assessment process. This empowered participants with the autonomy to reflect upon the workshop's content and independently gauge their learning. Notably, the moderator abstained from providing assessments or feedback throughout the workshop, ensuring a space for independent reflection and ownership of the learning experience.

The data captured through this workshop ^16^ was later reviewed retrospectively. The entire process of workshop was explained by the facilitator in the beginning. The participants were actively encouraged to ask questions and clarify any doubts from time to time specially before self-rating at various intervals. This helped reduce any potential bias while rating themselves. The participants provided informed consent when completing the self-administered rating questionnaire in writing. The study complied with tenets of the Declaration of Helsinki. The study involved the study participants’ perceptions and study approval was obtained from the Institutional Review Board (IRB) of the Institute Ethics Committee (IEC) Hyderabad Eye Research Foundation later with the Ethics Reference Number: LEC-BHR -01-23-151 approved on 22 ^nd^ March 2023.

The partcipants (Fellowship batch 2022, number=29) were initially invited via e-mail to participate in the presentation skills workshop.

The e-mail communication also included clear guidelines to prepare a 5-minute presentation for the workshop, on certain topics and presenting it in person during the workshop. The workshop began with a speech by the facilitator/moderator introducing the structure and sequential content, inviting the participants to fill-in a self-rating questionnaire survey. The self-assessment form was distributed to all partcipants and the self-rating procedure was explained to them at the beginning of the workshop. The self-ratings were administered at four different time points of the workshop (See Table 1); pre-workshop (after introduction), during the workshop (after trainee presentations), end of-workshop (after concluding workshop intervention) and final response on scope of improvement towards the end of the workshop (Post workshop intervention). The moderator allowed participant queries or suggestions, facilitating knowledge sharing.

**Table 1.** Description of self-rating questionnaire ^18^.

Serial Number	Self-rating questionnaire *	Time points
1	Rate your presentation skills (scale of 1–10) -properties and content (PC1) - soft skills (SS1)	Pre-workshop (after introduction) PC1, SS1
2	Rate your presentation skills today (scale of 1–10) - properties and content (PC2) - soft skills (SS2)	During the workshop (after trainee presentation) PC2, SS2
3	Rate your presentation skills today (scale of 1–10) - properties and content (PC3) - Soft skills (SS3)	At end of workshop intervention PC3,SS3
4	Rate your scope of improvement - properties and content (PC 4) - soft skills (SS4)	Post-workshop intervention PC4,SS4
5	Do you feel these workshops need to be revisited? Yes No	Post-workshop intervention
6	If YES: how frequently? Quarterly Biannually Annually	Post-workshop intervention

*Footnote: 1.Properties and Content (PC), and 2. Soft Skills (SS) at four different time points 1,2,3,4.

### Self-administered rating

The self-rating consisted of ten-point rating scales (1–10) which evaluated two main components of presentation skills, 1. Properties and Content (PC), and 2. Soft Skills (SS) at four different time points. The final response collected participant perceptions on improvement in presentation skills, commenting on and sharing their preference for content, duration, location, format, and frequency of participation in similar workshops. (See Table 1)

Properties & Content (PC) include presentation medium, alignment to broad guidelines of a PowerPoint presentation slide, audio visual aids, innovative demonstration objects and medium of presentation.

Soft Skills (SS) include Preparedness for the presentation, grooming, stage presence, body language & eye contact, voice projection active listening.

### Statistical analysis

Analyses were performed using SPSS Version 21.0 (IBM SPSS Statistics Inc., Armonk, NY, USA) software ^17^. Normality was checked using Shapiro Wilk test. Continuous data were represented as median range or interquartile range. Categorical data were presented in proportions. Multiple comparative analyses were performed using Friedman test. A probability value < 0.05 was considered statistically significant. Friedman test post-hoc analysis was applied for multiple comparisons and p-value < 0.02 was considered statistically significant.

## Results

**Figure 1.**
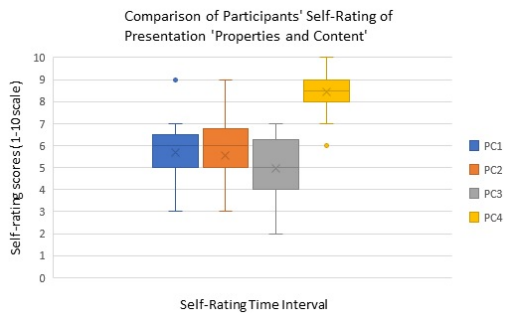
Box plot showing the comparison of participants’ presentation skills’ Properties and Content (PC) self-rating at different time points of the workshop. (PC1: Self rating after introduction, PC2: Self-rating during the workshop (after trainee presentation) PC3: Self-rating at end of workshop intervention, PC4: Self-rating at post-workshop intervention for gauging improvement).

The analyses of self-rating scores of ophthalmology fellows at four different time points are illustrated in Figure 1 (See Figure 1). In all, 29 participants in first year ophthalmology fellowship {19(65.51%) females and 10 (34.49%) males} were part of this workshop. The mean age of the participants was 28.89 +/- 2.5 (Mean, SD) years.

The box-whisker plot in Figure 1 (See Figure 1) demonstrates participant self-rating about ‘properties and content’ of their presentation skill on a scale of 1–10 (with one indicating minimum and ten the maximum rating of oneself in presentation skill acquisition) at four different time points of the workshop (PC1, PC2, PC3, PC4). For all participants, the self-rating at time point 4 (PC4), indicated improvement in ‘properties and content’ of the presentation compared to self-rating at the remaining three time points PC1, PC2, and PC3. This difference was statistically significant (Friedman Test, p<0.001). The median value at time point PC3 too showed that the participants rated lower on properties and content aspects of presentation skills compared to earlier time points PC1 and PC2, however it was statistically insignificant.

**Figure 2.**
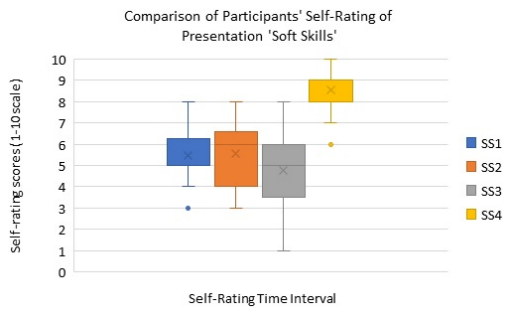
Box plot showing the comparison of self-rating of soft skills (SS) in presentation skills at four different time points of the workshop. SS1: self-rating after introduction to presentation skills workshop intervention, SS2: self-rating during the workshop (after trainee presentation), SS3: self-rating at end of workshop intervention, and SS4: self-rating for improvement post workshop intervention).

The box-whisker plot in Figure 2 (See Figure 2) demonstrates the self-rating results of participants on how they rate their learning of ‘soft skills’ during the presentation skills workshop on a scale of 1–10 at four different time points of the workshop (SS1, SS2, SS3, SS4). The self-rating 4 (SS4), scope of improvement in soft skills was found to be higher among participants compared to self-rating at SS1, SS2, and SS3. This difference was statistically significant (Friedman Test, p<0.001). The median value of SS3 shows that the participants’ rated lower on soft skill aspects of presentation compared to SS1 and SS2, however, it was statistically insignificant.

Overall, our study indicates that the presentation skills workshop positively impacted the participants, and 93.10% (27 ophthalmology fellows) preferred revisiting the workshop for biannual (33%) or quarterly sessions (33%).

## Discussion

While only a few studies have been published on impact of presentation skills training for ophthalmology students, there are several published articles focusing on presentation skills and communication skills of nurses and other health workers. A study conducted by Fowler and Jones that implemented an educational intervention to develop effective presentation skills in postgraduate nurses showed significant improvement in actual and perceived effectiveness of overall presentation skills ^19^. In our study too, the overall perceived effectiveness of workshop intervention in presentation skills was captured. The scope for improvement of the properties and content and soft skills components at fourth or last time point PC4 and SS4 revealed positive reactions to the presentation skills training. The corresponding f-test analyses (with Bonferroni assumption/Friedman post-hoc analyses) of participants’ self-ratings indicated that the training program led to higher scope for improvement in the participants’ presentation skills for the same components (PC4 and SS4) surveyed at the last time point.

Another study done by Seibold *et al* evaluated presentation skills training for participants from different industries (manufacturing, service, production, and research organizations) using a quasi-experimental design on behavioural skills ^20^. Statistical analyses showed positive reactions to the training, improvement in learning, short-term and long-term behavioural change, and on-the-job change in several skills. Similarly, in our study, the participant’s self-ratings indicated their perceived willingness to alter opinions and related behaviours that would demonstrably improve their personal presentation skills.

Our study showed lower mean scores of self-ratings PC1 and SS1 at the beginning of the workshop compared to the self-rating PC4 and SS4 post-workshop which could be because of greater awareness and shared learning during skill transfer in the workshop. As the workshop progressed, participants were able to reflect their presentation more objectively as evidenced by decline in self rating (PC3, SS3).

The workshop succeeded in providing the participants a structure for developing presentations. By fostering this skill set, ophthalmology fellows will be empowered to deliver impactful presentations in classrooms, at conferences, and on various other platforms, solidifying their development as astute young clinician-presenters. Each day, fellows participate in a dedicated hour of academic sessions led by our esteemed faculty. These sessions provide a vital platform for participants to hone their presentation abilities, a fundamental skill that underpins success in the field. The knowledge and practice gained through these workshops contribute significantly to the development of this core competency. Furthermore, we have integrated training in presentation skills into our immersive induction programs, ensuring that every incoming trainee batch receives a strong foundation in this essential skill.

Tutoring novice doctors in presentation skills during induction into a tertiary eye care setting would equip them to seize presentation opportunities for growing their experience and acumen. Each time the trainees face an audience, whether of peers, colleagues, co-workers, patients and their families, donors, institutional management boards, vendors, or other stakeholders, they are catapulting themselves of their own zealous accord closer to leadership orbits and satellite positions of centre stage power in eye care service delivery.

The acceptance of our workshop is evident from the positive comments received from participants’ feedback, requesting a periodic (quarterly/biannual) intervention on presentation skill ^21, 22^. We did not consider feedback on changing the workshop format as we have evolved from conducting virtual to hybrid format workshops over time. For greater participation, medical education is designed to include didactic lectures, simulated surgery, and increasingly, flipped or blended learning ^23– 26^. Transferring subject expertise through collaborative discussions requires dedicated time, so all these elements need to be carefully employed when conducting a short-term medical workshop intervention ^27– 29^.

## Feedback and recommendations

The rigor and frequency of these workshops are not given due importance, perhaps because the hands-on training is not immediately applied, or not practiced in the spare time that must be created during a busy clinical workday.

We assert that this presentation skills workshop intervention is required for learners in all phases of higher medical education ^30– 33^. Some of the ways to improve workshop effectiveness are having fewer participants in the workshop for a higher trainer: student ratio, using audiovisual aids for easier visualization of and guided explanation of the teaching points, and deploying teachable moments for riveting their attention ^34– 37^. These educational strategies might be more relevant for training learners who require special attention. We strongly recommend educators in ophthalmology to incorporate into the syllabus, actively teach and practice presentation skills to better help learners in all phases of their training. Workshops on presentation skills and related foundational skills, such as managerial, technical and leadership skills, may ingrain in tertiary care trainees the holistic perspective required for an effortless yet steep learning curve. We recommend schooling tertiary care trainees in observation, curiosity, critical thinking, and similar basic building blocks or foundational skills of professionalism be started from primary and secondary care levels for optimal proficiency in science and medicine ^38– 41^.

Curiosity, which kindles creativity and rolls out sharing and discussion as by-products, initiates innovation, discovery, and invention ^42^. Communication and dissemination take forward the outcomes of curiosity – products, processes, and knowledge – to time-spaces near and far, for doing greater public good. Empathy, the basis of communication, builds the bonds that integrate all levels of bonding in society and is reported to decline in medical training ^43, 44^. After teasing out the technicalities of presentation skills, such as knowledge of the features of Microsoft PowerPoint, dealing with a breakdown midway, or maintaining a rapid pace of presentation, what remains are the creative and empathic factors in professional communication ^45^. Nonverbal communicativeness (by adopting measures such as proffering eye contact and a half smile that reaches the eyes, for presenting oneself well) signals the credibility of the speaker and topic, and can be medicinal, mood-elevating, and health-giving. Every effort must be made to inculcate empathy in medical trainees, so that they present well not only themselves but also the legacy institutions, mates, and others that they represent, in hospital corridors and wider world circles.

While being master (and slave) of the digital technology (multimedia production and display) of our times, continuing the presentation with personable equanimity (by staying unruffled on occasions when team spirit, system, software, or power supply become dysfunctional) is the ultimate test of one’s empathic leadership. We believe that training may be maximized by encouraging participants to speak publicly and share widely their subject expertise – in the format of their recently acquired presentation skills ^46^. Along with such truly generous knowledge sharing, the surgeons’ attentiveness, learning and doing may disrupt disparities, hastening translational research in our society and razing the inequities miring our world ^47^. The movement back and forth from presentation granularity afforded by quality checklists to presenter self-reflexivity may work wonders for our post-pandemic digital endeavours – deep breathing life into our humanity and symbiotic sociality.

## Limitations

- The self-reported data may be biased.
- Long term assessment of the skill has not been incorporated in the training plan
- Sample size may limit the generalizability of the reported outcomes.

## Data Availability

Figshare: Presentation skills - Data .csv. https://doi.org/10.6084/m9.figshare.25138109 ^16^ This project contains the following underlying data: Presentation skills - Data .csv Figshare: Presentation Skill (self rating) questionnaire. https://doi.org/10.6084/m9.figshare.25231448 ^18^ Figshare: Participants' Feedback. https://doi.org/10.6084/m9.figshare.25887388.v1 ^21^ Figshare: Revisiting workshop data https://doi.org/10.6084/m9.figshare.25895062.v1 ^22^ This project contains the following extended data: Presentation Skill (self rating).docx Data are available under the terms of the Creative Commons Zero "No rights reserved" data waiver (CC0 1.0 Public domain dedication).
