# Supplementary material for: Experience of focused workshop intervention in presentation skills - Importance of foundational skills for ophthalmologists in training
Source: MedEdPublish (2016). 2024 May 29;14:18. Originally published 2024 Mar 27. [Version 2] doi: 10.12688/mep.20114.2 (PMC11224709; doi:10.12688/mep.20114.2)
Supplement: Peer review history

# Reviewer report: Reviewer response for version 2

**DOI:** 10.21956/mep.21903.r37258

**Published:** 2024-07-04

**Authors:** Ninani Kombo

**Affiliations:** 1 Yale School of Medicine, Connecticut, USA

Thank you for incorporating your edits. I have a few more suggestions. Once you incorporate these it is complete.

 Results section in the abstract

 Remove & and write ‘and’

 Introduction

 In the second paragraph of the introduction, leave out ‘for professional presenter’ and start the paragraph with Presentation skills……

 Methods

 ‘for fellows under training pursuing an ophthalmology fellowship can be shortened to read ‘for ophthalmology fellows’

 Second sentence can be rewritten as such ‘All participants (29) were recruited from fellowship participants. Study Eligibility was limited to current fellows. 

 Third sentence ‘ The workshop was conducted by a facilitator in a classroom setting with the use of audiovisual technology. ’

 We can leave out the last sentence

 I recommend that the 4 ^th^ and 5 ^th^ paragraph be moved to be the first of the methods section. It clarifies the methods and sets the tone. The first sentence of the 4th paragraph can remain at the end.

 Results – great

 Discussion

 Paragraph 1 While only a few studies have been published on **the **impact…..for ophthalmology **trainees**,

 Paragraph 4 The workshop succeeded in providing the participants **with** a structure…..

 Limitation

 Point 2 long term assessment of the **skills** have not been incorporated into the training plan.

Have any limitations of the research been acknowledged?

Yes

Is the work clearly and accurately presented and does it cite the current literature?

Yes

If applicable, is the statistical analysis and its interpretation appropriate?

Yes

Are all the source data underlying the results available to ensure full reproducibility?

Yes

Is the study design appropriate and is the work technically sound?

Yes

Are the conclusions drawn adequately supported by the results?

Yes

Are sufficient details of methods and analysis provided to allow replication by others?

Yes

Reviewer Expertise:

Medical Education

I confirm that I have read this submission and believe that I have an appropriate level of expertise to confirm that it is of an acceptable scientific standard.

# Reviewer report: Reviewer response for version 2

**DOI:** 10.21956/mep.21903.r37259

**Published:** 2024-06-03

**Authors:** Chen Zhi Xiong

**Affiliations:** 1 Yong Loo Lin School of Medicine, National University of Singapore, Singapore, Singapore

The authors have revised the manuscript to a reasonable extent and provided additional data to support the findings and recommendations. I wish the authors well in implementing similar interventions across topics and disciplines.

Have any limitations of the research been acknowledged?

No

Is the work clearly and accurately presented and does it cite the current literature?

Partly

If applicable, is the statistical analysis and its interpretation appropriate?

I cannot comment. A qualified statistician is required.

Are all the source data underlying the results available to ensure full reproducibility?

Partly

Is the study design appropriate and is the work technically sound?

Partly

Are the conclusions drawn adequately supported by the results?

Partly

Are sufficient details of methods and analysis provided to allow replication by others?

Partly

Reviewer Expertise:

NA

I confirm that I have read this submission and believe that I have an appropriate level of expertise to confirm that it is of an acceptable scientific standard.

# Reviewer report: Reviewer response for version 1

**DOI:** 10.21956/mep.21539.r36775

**Published:** 2024-05-21

**Authors:** Ninani Kombo

**Affiliations:** 1 Yale School of Medicine, Connecticut, USA

A great article addressing presentation education in the field of ophthalmology. Impressive that this short intervention had such wonderful results.

 My comments and recommendations are as follows:

 in the methods section change 'trainees' to fellows.

 one suggestion is to have the methods section in the abstract written as such:

 A 10-hour workshop on presentation skills was conducted for ophthalmology fellows in the 2022 cohort at a single institution. An email was sent to the 2022 cohort to recruit participants. A total of 29 fellows (19 females, 10 males) volunteered to participate in the study. 'And then continue with what you have written for the rest of the methods.

 I recommend grammar, and sentence structure editing throughout the paper to improve readability.

 Try to keep the writing more technical. 

Have any limitations of the research been acknowledged?

Yes

Is the work clearly and accurately presented and does it cite the current literature?

Yes

If applicable, is the statistical analysis and its interpretation appropriate?

Yes

Are all the source data underlying the results available to ensure full reproducibility?

Yes

Is the study design appropriate and is the work technically sound?

Yes

Are the conclusions drawn adequately supported by the results?

Yes

Are sufficient details of methods and analysis provided to allow replication by others?

Yes

Reviewer Expertise:

Medical Education

I confirm that I have read this submission and believe that I have an appropriate level of expertise to confirm that it is of an acceptable scientific standard, however I have significant reservations, as outlined above.

# Reviewer report: Reviewer response for version 1

**DOI:** 10.21956/mep.21539.r36776

**Published:** 2024-05-09

**Authors:** Chen Zhi Xiong

**Affiliations:** 1 Yong Loo Lin School of Medicine, National University of Singapore, Singapore, Singapore

Communication is a key aspect of professionalism. Interventions such as workshop can be effective in training communication. If facilitated skilfully, it may foster curiosity and empathy as an outcome if that is the intent. This is a good piece of work on the importance of training communication in ophthalmology through a single 10-hour workshop intervention. Below are comments and suggestions that may strengthen this work.

1. Whether a workshop is effective as an intervention depends on what goes on in the workshop, in this regard, please provide more details on the workshop under ‘Methods’, specifically,
  a. Was feedback provided to participants after their presentations?
  b. If feedback were given, were participants given a chance to present again?
2. Was qualitative feedback obtained? If yes, it will be good to show this data.
3. Please provide the definitions of ‘properties and content (PC)’ and ‘soft skills (SS)’ under ‘Methods’.
4. The workshop made participants aware of their areas for improvement. In this regard, please discuss whether there are plans to develop the participants further. 5. Please include limitations of the study.

Minor:

1. In the ‘Results’ under ‘Abstract’, it is inaccurate to state “…presentation skills PC and SS significantly improved…” as the higher post-workshop rating is in the context of ‘scope of improvement’, suggesting that participants were made aware of their areas for improvement, rather than actual improvement.
2. Under ‘Introduction’, it may be better to focus on patients, rather than doctors’ “aspirations” or popularity.
3. Though insignificant, please discuss why PC3 and SS3 may be lower than PC1 and PC2, and SS1 and SS2 respectively.
4. Under ‘Discussion’, please amend to “Our study showed the *lower* mean scores of self-ratings PC1 and SS1 at the beginning of the workshop *compared* to the…”.
5. Please indicate when PC4 and SS4 was obtained after the workshop, i.e. how long after the workshop.
6. Under ‘Discussion’, it was mentioned “The workshop succeeded in providing the participants a structure for developing both written and oral presentations.”. However, the study appears to focus on oral presentations only. Please clarify or amend.
7. Please provide data for questionnaire serial number 5 and 6 where available.
8. Please simplify the language under ‘Discussion’ and ‘Feedback and recommendations’.

Have any limitations of the research been acknowledged?

No

Is the work clearly and accurately presented and does it cite the current literature?

Partly

If applicable, is the statistical analysis and its interpretation appropriate?

I cannot comment. A qualified statistician is required.

Are all the source data underlying the results available to ensure full reproducibility?

Partly

Is the study design appropriate and is the work technically sound?

Partly

Are the conclusions drawn adequately supported by the results?

Partly

Are sufficient details of methods and analysis provided to allow replication by others?

Partly

Reviewer Expertise:

NA

I confirm that I have read this submission and believe that I have an appropriate level of expertise to confirm that it is of an acceptable scientific standard, however I have significant reservations, as outlined above.
